# Hyperpolarized ^13^C pyruvate magnetic resonance spectroscopy for in vivo metabolic phenotyping of rat HCC

**DOI:** 10.1038/s41598-020-80952-4

**Published:** 2021-01-13

**Authors:** Elisabeth Bliemsrieder, Georgios Kaissis, Martin Grashei, Geoffrey Topping, Jennifer Altomonte, Christian Hundshammer, Fabian Lohöfer, Irina Heid, Dominik Keim, Selamawit Gebrekidan, Marija Trajkovic-Arsic, AM Winkelkotte, Katja Steiger, Roman Nawroth, Jens Siveke, Markus Schwaiger, Marcus Makowski, Franz Schilling, Rickmer Braren

**Affiliations:** 1 School of Medicine, Institute of Diagnostic and Interventional Radiology, Technical University of Munich, 81675 Munich, Germany; 2 School of Medicine, Department of Nuclear Medicine, Technical University of Munich, 81675 Munich, Germany; 3 School of Medicine, Clinic and Policlinic of Internal Medicine II, Technical University of Munich, 81675 Munich, Germany; 4 Division of Solid Tumor Translational Oncology, German Cancer Consortium (DKTK, Partner Site Essen) and German Cancer Research Center, DKFZ, Heidelberg, Germany; 5 Institute for Developmental Cancer Therapeutics, West German Cancer Center, University Medicine Essen, 45147 Essen, Germany; 6 School of Medicine, Institute of Pathology, Technical University of Munich, 81675 Munich, Germany; 7 School of Medicine, Clinic and Policlinic of Urology, Technical University of Munich, 81675 Munich, Germany; 8 Department of Computing, Imperial College London, London, SW7 2AZ UK

**Keywords:** Molecular medicine, Diagnostic markers, Biomarkers, Preclinical research, Translational research

## Abstract

The in vivo assessment of tissue metabolism represents a novel strategy for the evaluation of oncologic disease. Hepatocellular carcinoma (HCC) is a high-prevalence, high-mortality tumor entity often discovered at a late stage. Recent evidence indicates that survival differences depend on metabolic alterations in tumor tissue, with particular focus on glucose metabolism and lactate production. Here, we present an in vivo imaging technique for metabolic tumor phenotyping in rat models of HCC. Endogenous HCC was induced in Wistar rats by oral diethyl-nitrosamine administration. Peak lactate-to-alanine signal ratios (L/A) were assessed with hyperpolarized magnetic resonance spectroscopic imaging (HPMRSI) after [1-^13^C]pyruvate injection. Cell lines were derived from a subset of primary tumors, re-implanted in nude rats, and assessed in vivo with dynamic hyperpolarized magnetic resonance spectroscopy (HPMRS) after [1-^13^C]pyruvate injection and kinetic modelling of pyruvate metabolism, taking into account systemic lactate production and recirculation. For ex vivo validation, enzyme activity and metabolite concentrations were spectroscopically quantified in cell and tumor tissue extracts. Mean peak L/A was higher in endogenous HCC compared to non-tumorous tissue. Dynamic HPMRS revealed higher pyruvate-to-lactate conversion rates (*k*_*pl*_) and lactate signal in subcutaneous tumors derived from high L/A tumor cells, consistent with ex vivo measurements of higher lactate dehydrogenase (LDH) levels in these cells. In conclusion, HPMRS and HPMRSI reveal distinct tumor phenotypes corresponding to differences in glycolytic metabolism in HCC tumor tissue.

## Introduction

Hepatocellular carcinoma (HCC) is the sixth leading cause of cancer-related death worldwide, with over 780,000 deaths in 2018 and over 840,000 new cases according to the Global Cancer Observatory^1^. Genetic and proteomic heterogeneity represent obstacles to therapy, limiting therapeutic choices in advanced stages^2^ and rendering resection or transplantation the sole curative options. Recently, metabolic heterogeneity, i.e. the differential utilization of metabolites (mainly glucose) in tumor tissues, has entered the spotlight as a potential diagnostic and therapeutic endpoint. The glucose metabolite lactate plays a central role in several tumor entities by promoting neoangiogenesis, tumor invasion and immune evasion^3–6^. For example, head and neck tumors with high lactate production demonstrate a more aggressive phenotype^7–9^. In HCC, recent evidence supports the notion of different metabolic subtypes^10^, which correlate with patient outcomes. This has been linked to alterations in concrete metabolic pathways and led to the testing of novel therapeutic interventions aimed at such endpoints^11^.

Due to multifocality and multimorbidity (in particular coagulopathy), biopsies are not always an option for the diagnosis or the longitudinal monitoring of HCC. Current non-invasive imaging methods such as positron emission tomography (PET) can visualize and quantify the uptake of the glucose analogue ^18^F-fluordesoxyglucose (^18^F-FDG) in tumor tissues, but do not permit the measurement of downstream metabolite utilization. Furthermore, although the uptake of ^18^F-FDG has been used for the prediction of tumor grading in both humans and translational model systems and correlates with clinical outcome in HCC patients^12^, PET’s sensitivity in HCC detection is limited by elevated glucose uptake in the surrounding liver tissue in patients with chronic liver disease^13^.

Magnetic resonance spectroscopy and spectroscopic imaging (HPMRS(I)) after hyperpolarized [1-^13^C]pyruvate injection are techniques for the real-time measurement of metabolic turnover, more specifically of lactate dehydrogenase (LDH) activity. Dynamic nuclear hyperpolarization enhances the nuclear magnetic resonance (NMR) signal by four to five orders of magnitude, overcoming the limited sensitivity of conventional (thermally polarized endogenous signal) MRS(I)^14^ and the low natural abundance of the MR-active ^13^C isotope in vivo. Pyruvate is particularly well suited for metabolic imaging, due to it being the end product of glycolysis and metabolized to alanine or lactate^15,16^. Measurable changes in pyruvate conversion occur within [1-^13^C]pyruvate’s longitudinal relaxation time (*T*_1_, 30–40 s), making it suitable for in vivo hyperpolarized imaging^17^. In comparison to PET, hyperpolarized MRSI achieves similar sensitivity for early therapy response assessment^18^.

We here present the application of HPMRS(I) in a clinically relevant translational HCC model and demonstrate its potential for the non-invasive subtyping of tumors based on detection and quantification of pyruvate utilization.

## Material and methods

### Animal models and preparation

Animal experiments were approved by the local governmental committee for animal protection and welfare (Tierschutzbehörde Regierung von Oberbayern, Protocol Nr. 55.2-1-54-2532-25-2016). All procedures were carried out in accordance with applicable laws and regulations. Two animal cohorts were employed in the study. Cohort A included 17 male Wistar rats (RccHan:WIST; six to eight weeks old; Envigo; Re Schaijk, Netherlands), consisting of 13 rats in which HCCs were induced by oral administration of 0.01% diethyl-nitrosamine (DENA, SigmaAldrich) dissolved in drinking water over ten weeks, as previously described^19^, and 4 healthy control rats. Cohort B consisted of ten male nude rats (Crl:NIH-Foxn1^rnu^; six to eight weeks old; Charles River, Sulzfeld, Germany) in which subcutaneous tumors were implanted in each flank. Tumor screening in cohort A rats was performed by *T*_2_-weighted (*T*_2w_) anatomical imaging on a human 3 T clinical MRI system (Philips Ingenia 3.0 T; Philips Medical, Amsterdam, Netherlands) as previously described^12^. Tumor screening in cohort B rats was performed by caliper measurement. Tumor bearing animals were included in HPMRS(I) experiments once tumors reached ≥ 10 mm in diameter.

### Tumor cell lines

Cells were isolated from endogenous tumor tissue of cohort A animals by homogenization in cell culture media (described below). The suspension was incubated (37 °C; 30 min) with Liberase TM (La Roche, Basel, Switzerland), filtered through a 100 µm cell filter (BD Bioscience, Franklin Lakes, USA) and centrifuged (500 g; 5 min). After reaching confluence, cells were split 1:100. After single clones arose, they were separated with trypsin drenched filter paper and transferred to 24-well plates for expansion. Two cell lines, isolated from a low and a high lactate/alanine ratio tumor, were used for further experiments. These two cell lines (low: LA-L and high: LA-H) were expanded and frozen down at early passage. 1 × 10^6^ tumor cells were subcutaneously implanted in each flank of Cohort B-animals.

### In vitro experiments on tumor cell lines

Cells were cultivated in Dulbecco’s modified Eagle Medium (DMEM) containing L-glutamine (Biowest, Nuaillé, France), 10% fetal calf serum (Merck, Darmstadt, Germany), 1% sodium pyruvate (Merck, Darmstadt, Germany), 1% non-essential amino acids (GE Healthcare, Chicago, USA) and 1% penicillin and streptomycin (PAN Biotech, Aidenbach, Germany) at 37 °C with 5% CO_2_. LDH activity assays were performed in triplicates. 10^6^ cells were lysed in radioimmunoprecipitation assay (RIPA) buffer (1 M Tris–HCl (pH 8), 1 M NaCl, Nonidet P40, sodium deoxycholate, distilled water) and after centrifugation (4 °C; 5000 rpm; 15 min), LDH activity in the supernatant was determined photometrically (cobas c 701/702 system, Roche/Hitachi; LDH-activity: LDHI2 (Lactate Dehydrogenase acc. to IFCC ver.2), Hoffmann—La Roche, Basel, Switzerland) following manufacturer’s instructions. Metabolic flux was measured using the *Seahorse* assay, for which cells were cultivated in DMEM media supplemented with 5 mM glucose, 4 mM glutamine, 10% FBS and 1% Pen/Strep. Real time measurement of the extracellular acidification rate was performed on a *Seahorse* XFe96 analyzer (Agilent Technologies Inc., Santa Clara, USA) using a *Seahorse* XF glycolysis stress test kit (Agilent Technologies Inc., Santa Clara, USA) following manufacturer’s instructions, with 10^4^ cells per well in a 96 well plate in culture media. After the assay, the cells were fixed with 4% PFA and stained with DAPI nuclear dye. Fluorescence was assessed using the XFe96 analyzer and used for normalization of the *Seahorse* XF data (RFU, relative fluorescence unit normalization).

### Ex vivo experiments on primary and transplanted tumor extracts

After euthanasia, tumors were removed rapidly and divided into two parts. One part was formalin-fixed for histopathological analyses, and the other was frozen in liquid nitrogen and kept at -80 °C for further analyses.

For enzyme activity measurements, 90–110 mg of tumor tissue were homogenized in 1 ml RIPA buffer (FastPrep-24 Tissue and Cell Homogenizer, MP Biomedicals, Santa Ana, USA). After centrifuging, lactate dehydrogenase (LDH) and/or glutamate pyruvate transaminase (GPT) activity were determined photometrically (cobas c 701/702 system, Roche/Hitachi; LDH-activity: LDHI2 (Lactate Dehydrogenase acc. to IFCC ver.2) and GPT-activity: ALTPM (Alanine Aminotransferase acc. to IFCC with pyridoxal phosphate activation), Hoffmann—La Roche, Basel, Switzerland) following manufacturer’s instructions.

For ^1^H NMR spectroscopy of metabolite concentrations, 50–120 mg samples of tumor tissue were homogenized in 4 ml/g methanol and 0.85 ml/g distilled water. After homogenization, 4 ml/g chloroform and 2 ml/g distilled water were added, mixed, incubated on ice (10 min) and centrifuged (4 °C; 2000 g; 5 min). The polar phase of the probe was removed for lyophilization (Alpha 1–2 LDplus, Martin Christ Gefriertrocknungsanlagen GmbH, Osterode am Harz, Germany). Freeze-dried specimens were resuspended in 590 μl distilled water and 10 μl deuterium. The proton NMR spectra were measured in a 300 MHz NMR spectrometer (7.04 T, Avance III HD 300 MHz Vertical Bore Spectrometer, TUM chemistry department, Bruker Biospin, Germany) with a pulse-acquire sequence (16 scans + 2 dummy scans; flip angle 30°; repetition time (TR) 6.45 s; receive bandwidth 6 kHz; acquisition time 5.45 s; FID-size 65,536 points; total scan time 1 min 56 s). For lactate and alanine quantification in tumor extracts, integrals over the assigned peak doublets of each metabolite in the phased and baseline-corrected spectra were compared to the integral of 0.01 mM hydroquinone. (Supplementary Figure S1). To determine absolute concentrations, the arithmetic sum of the 0.01 mM hydroquinone reference spectrum and the spectra of the probes were calculated with MNOVA (MestReNova, 10.0 Mestrelab Research, Santiago de Compostela, Spain).

### Hyperpolarization of [1-^13^C]pyruvate

For hyperpolarization of pyruvate, a 30 mg mixture containing 14 M [1-^13^C]pyruvate (Merck, Darmstadt, Germany), 16 mM OX063 (Oxford Instruments, Abingdon, UK) and 1 mM Gadoterate Meglumine (Dotarem, Guerbet Laboratories Ltd., Villepinte, France) was polarized with a HyperSense DNP Polarizer (Oxford Instruments, Abingdon, UK) using a microwave frequency of 94.172 GHz until 90% of the saturation polarization level at 3.35 T and 1.2 K was reached. The frozen sample was rapidly dissolved using 4.2 ml buffer solution containing distilled water, 80 mM TRIS (Merck, Darmstadt, Germany), 0.1 g/l EDTA (Merck, Darmstadt, Germany) and 80 mM sodium hydroxide (NaOH), which was preheated to 180 °C resulting in an 80 mM [1-^13^C]pyruvate solution at a physiological pH with a mean polarization level of 38% at the start of dissolution under the given experimental conditions^20^.

### Magnetic resonance spectroscopy and imaging

Rats were anesthetized by isoflurane (1.5–2.5%; O_2_-flow: 2 l/min; CP-Pharma, Burgdorf, Germany) and tail vein catheterized (Becton Dickinson Company, Franklin Lakes, USA) for the injection of the hyperpolarized agent. Animals were kept at 37 °C using warm air (Mistral-Air Plus, The37°Company, Amersfoort, Netherlands) and temperature and breathing were monitored with a rectal temperature sensor and pressure pad (SA Instruments Inc., New York, USA). Experiments were performed in a 7.0 T magnet (Agilent Technologies, Oxford, UK) small animal MRI scanner (initially Discovery MR901, GE Healthcare, Waukesha, Wisconsin, USA for hyperpolarized imaging; converted to AVANCE III HD electronics, Bruker Corporation, Billerica, USA for hyperpolarized spectroscopy). Anesthesia duration was kept consistent at an average of 95 min for all experiments to avoid differences in measurements due to anesthesia time.

For endogenous HCC, a 2D phase-encoded free induction decay chemical shift imaging (FID-CSI) sequence and a dual-tuned ^1^H/^13^C volume resonator (72 mm inner diameter, RAPID Biomedical GmbH, Würzburg-Rimpar, Germany) were used for radiofrequency transmission and signal reception. Metabolite images were acquired ~ 15 s after injection for 30 s with 9.9 s per single frame; matrix size 12 × 12; centric acquisition order in k-space; single slice thickness 5 mm; field of view 48 × 48 mm; receive bandwidth 5 kHz; 2048 spectral points; TR 68 ms; 6° flip angle; three time-frames. A total of 17 tumors in 13 DENA-treated rats and 4 healthy livers in 4 control rats were examined. Of these, 1 tumor scan was excluded due to a poor signal-to-noise ratio and shimming artefacts which made peak identification impossible.

For subcutaneous tumors, signal was measured by a 20 mm ^13^C surface receive coil (RAPID Biomedical GmbH, Würzburg-Rimpar, Germany) in combination with the ^1^H/^13^C volume resonator used for ^13^C transmission. A dynamic, slice-selective spectroscopy sequence with the following parameters was applied: 8 kHz transmit bandwidth, 2 kHz receive bandwidth; 512 acquisition points; center frequency between [1-^13^C]pyruvate and [1-^13^C]lactate at 175 ppm; TR 2 s; 5° flip angle and a single slice of thickness of 10 mm. Data acquisition began upon dissolution and 90 spectra were acquired over 180 s. Slice positioning was based on anatomical *T*_2w_ coronal and axial images (coronal: field of view 128 × 72 mm; TR 3 s; TE 20 ms; slice thickness 1 mm; data matrix 256 × 144; 36 slices; readout bandwidth 200 kHz; transmit bandwidth 2 kHz; axial: field of view 72 × 54 mm; TR 4 s; TE 48 ms; slice thickness 2 mm; data matrix 240 × 180; 35 slices; readout bandwidth 200 kHz; transmit bandwidth 2 kHz).

### ^13^C-data analysis

Data processing was performed in Matlab (The Mathworks, Inc., Natick, USA) with in-house developed software. FID-CSI HPMRSI data of endogenous HCCs (cohort A) were linearly interpolated in image size by a factor of two and line broadened by 40 Hz. Data was analyzed by the calculation of [1-^13^C]lactate to alanine peak signal ratios (L/A) from 2D whole tumor ROIs drawn based on the *T*_2w_ anatomical image. Magnitude spectral data of dynamic HPMRS-data (cohort B) was line-broadened by 30 Hz. Signal maxima of [1-^13^C]pyruvate and [1-^13^C]lactate were normalized to the maximum intensity of pyruvate. A fitting routine was developed based on a two-site kinetic exchange model as described in^21^. Incorporating back-conversion from lactate to pyruvate, *T*_1_-relaxation and RF-excitation losses into an effective decay rate *ρ*_pyr_, the pyruvate signal curve is given by

1 $${M}_{pyr}\left(t\right)= {M}_{pyr,max}\bullet {e}^{{-R}_{pyr,eff}\bullet t}$$

where the overall effective decay rate ${R}_{pyr,eff}= {k}_{pl}+ {\rho }_{pyr}$ is the sum of the kinetic pyruvate-to-lactate conversion rate constant *k*_pl_ and the effective decay rate ${\rho }_{pyr}$. Assuming back-conversion to be negligible, one can correct the effective spin–lattice relaxation constant ${T}_{1.pyr}^{*}=\frac{1}{{\rho }_{pyr}}$ for RF excitation losses by

2 $$\frac{1}{{T}_{1,pyr}}= \frac{1}{{T}_{1,pyr}^{*}}+\frac{ln(coscos \left(FA\right) )}{TR}$$

where *FA* is the excitation flip angle, *TR* the repetition time and ${T}_{1,pyr}$ the intrinsic spin–lattice constant of [1-^13^C]pyruvate in vivo at 7 T. Modelling of several datasets (see examples in Fig. 4, Supplementary Figure S2) yielded *k*_pl_ > *R*_pyr,eff_, hence giving *T*_1,pyr_ < 0 which is physically unreasonable^22^. The detected lactate signal in the tumor was therefore assumed to be augmented by inflowing lactate produced in other tissues (e.g. heart, liver, contralateral tumor) or by re-circulating lactate from the observed tumor itself. This observation was included in the model by assuming the time curve of inflowing lactate to be of the same shape as the time curve of overall lactate measured in the tumor, and therefore proportional to it. Mathematically, this was accounted for by separating the overall lactate signal into an inflow- and a tumor-originating fraction. The signal from tumor-originating lactate ${M}_{lac,tumor}$ is given by

3 $${M}_{lac,tumor}=SF\bullet {M}_{lac,tot}$$

with *SF* being a scaling factor between 0 and 1 (see the Supplementary Material for details). The lactate time curve from the tumor was then modelled according to

4 $$\frac{{dM}_{lac,tumor}}{dt}= {k}_{pl}\bullet {M}_{pyr}\left(t\right)-{R}_{lac,eff}\bullet {M}_{lac,tumor}\left(t\right),$$

which can be discretized and from which *k*_*pl*_ and *R*_*lac,eff*_ can be obtained using the Moore–Penrose pseudoinverse matrix. Here, *R*_*lac,eff*_ is the overall effective decay rate of the hyperpolarized lactate signal, including back-conversion from lactate to pyruvate, RF excitation and intrinsic *T*_1_ relaxation.

The scaling factor *SF* was chosen such that *T*_1,pyr_ = 30 s holds, derived from Eq. (2), based on previously reported values for *T*_1_ of [1-^13^C]pyruvate from in vivo experiments and taking into account the *B*_0_-field dependence of *T*_*1*_^22,23^. Quality-of-fit was evaluated based on the calculation of L1-residuals with a threshold of ~ 0.7 and visual inspection of fit quality at early timepoints.

### Histology and immunohistochemistry

Tumor cell agarose pellets were prepared from tumor cell lines, fixated in 10% neutral buffered formalin, paraffin embedded, sliced and confirmed as HCC by immunostaining for CK7 (dilution 1:200; Abcam) and HepPar1 (dilution 1:50; Dako)^24,25^.

The tumor specimens were formalin-fixed and paraffin-embedded according to standard protocol. The endogenous HCCs were stained with H&E for classification as HCC lesions by a board-certified veterinary pathologist (KS). The subcutaneous tumors were stained with H&E and anti-CD31 rabbit antibody (dilution 1:50; Abcam, Cambridge, UK) using an automated BondRxm staining unit. To analyze CD31 staining, 6 representative visual fields (20 × magnification) were allocated over the whole slide of each tumor, and vessel ingates were enumerated and then averaged.

### Statistical analysis

All statistical analyses were performed using Prism 7 (GraphPad Software, LaJolla, USA). The distribution of the values was tested with D'Agostino & Pearson normality test. For group comparison, non-normal data were analyzed with the Mann–Whitney-U and normally distributed data with the Student’s T-test. All data presented as mean with standard deviation. A statistical significance level of p < 0.05 was defined.

## Results

**Figure 1:**
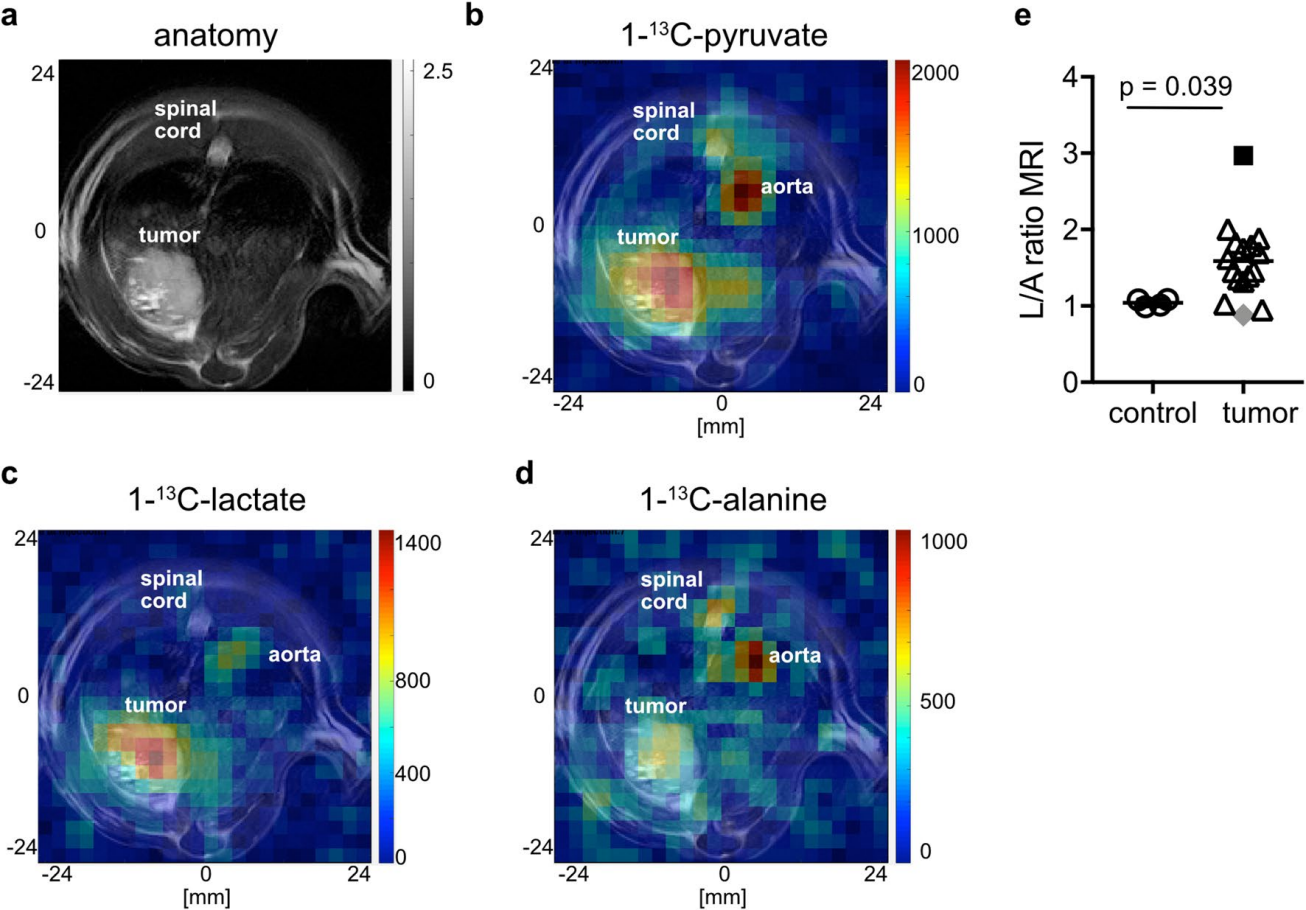
Example images of anatomy (*T*_2w_) and HPMRSI measurements overlaid atop anatomical *T*_2w_ images (linearly upscaled by 2 × to 24 × 24 matrix in-plane, color scale in arbitrary units but proportional between metabolite images) of tumor bearing animals. (**a**) axial liver anatomy, (**b**) [1-^13^C]pyruvate, (**c**) [1-^13^C]lactate and (**d**) [1-^13^C]alanine signal images. (**e**) Plot showing distribution of peak L/A of control liver and tumor tissue. Mann–Whitney-U-test p = 0.039. The tumors from which cells were isolated are denoted by a black square (LA-H) and a grey rhombus (LA-L).

To examine changes in the signal of each metabolite and therefore the glycolytic flux during HCC pathogenesis, FID-CSI after hyperpolarized [1-^13^C]pyruvate injection was performed in an endogenous HCC model and healthy control rats (Fig. 1a–d). HPMRSI revealed significantly higher L/A in tumor tissue (n = 16, mean_tumor_ 1.60 ± 0.50) compared to control animal normal liver tissue (n = 4, mean_control_ 1.04 ± 0.05; Mann–Whitney-U-test p = 0.039). Additionally, tumor L/A ratios were overdispersed, indicating differences in the metabolic attributes of the tumors (Fig. 1e).

**Figure 2:**
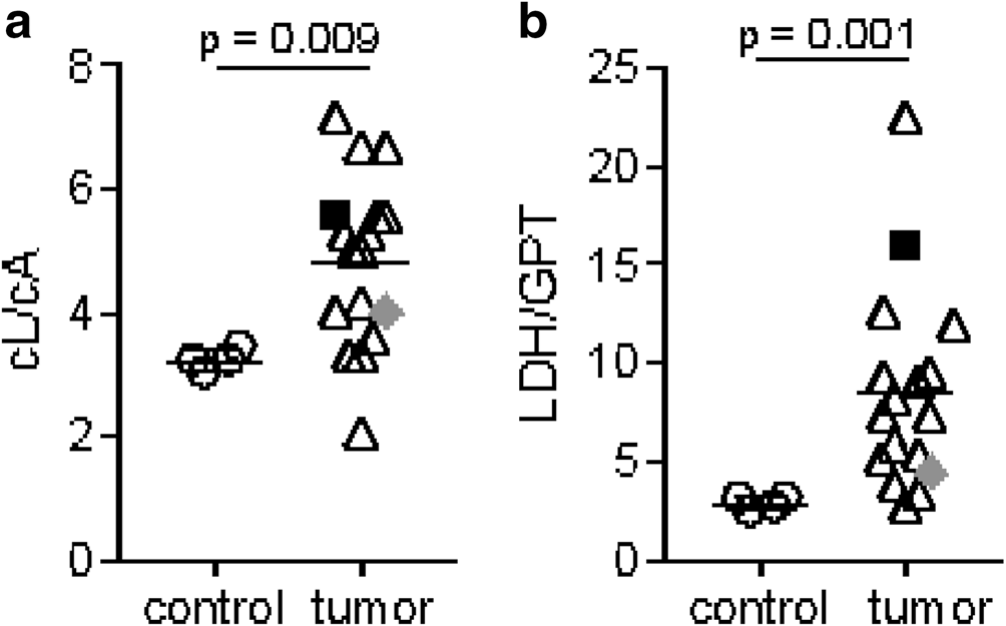
Ex vivo analysis of endogenous tumors and control livers of cohort A animals. L/A concentration ratios measured by ^1^H spectroscopy (**a**) and LDH/GPT activity ratios of tissue extracts (**b**) were compared using the Mann–Whitney-U test (p = 0.009 and p = 0.001, respectively).

Ex vivo validation confirmed these findings, showing both significantly elevated absolute L/A concentration ratios, due to significantly increased lactate pools (Supplementary Figure S1), and LDH/GPT ratios in tumor tissue extracts compared to normal liver extracts in ^1^H spectroscopy (Fig. 2a, mean_tumor_ 4.83 ± 1.36, n = 17; mean_control_ 3.24 ± 0.17; n = 4; Mann–Whitney-U-test-p = 0.009) and in enzyme activity assays (Fig. 2b, mean_tumor_ 8.48 ± 5.04, n = 17; mean_control_ 2.81 ± 0.33; n = 4; Mann–Whitney-U-test-p = 0.001). Examples of spectra of control (a) and tumor (b) tissue are shown in Supplementary Figure S1. The determined metabolite ratios from ^1^H spectroscopy were also used to assess the influence of the steady state metabolite pool sizes from lactate and alanine on the metabolite ratios obtained from hyperpolarized ^13^C MRSI imaging. Metabolite ratios from imaging (L/A ratio 13C-MRI) and ^1^H spectroscopy (cL/cA ^1^H MRS) showed no considerable correlation (see Supplementary Figure S3), indicating that the total metabolite pool size obtained from 1H MRS does not strongly influence the observed in vivo metabolite kinetics in our model.

To further assess the role of lactate in HCC, primary tumor cell lines were isolated and characterized from two tumors showing the lowest and highest L/A in HPMRSI (see Fig. 1e) before reimplantation (LA-L and LA-H cells). Both cell lines were confirmed as HCC clones by immunostaining for HepPar1 and CK7. LA-H cells exhibited a pleomorphic partially elongated shape compared to the more compact, cuboid shape of LA-L cells (see Supplementary Figure S4).

**Figure 3:**
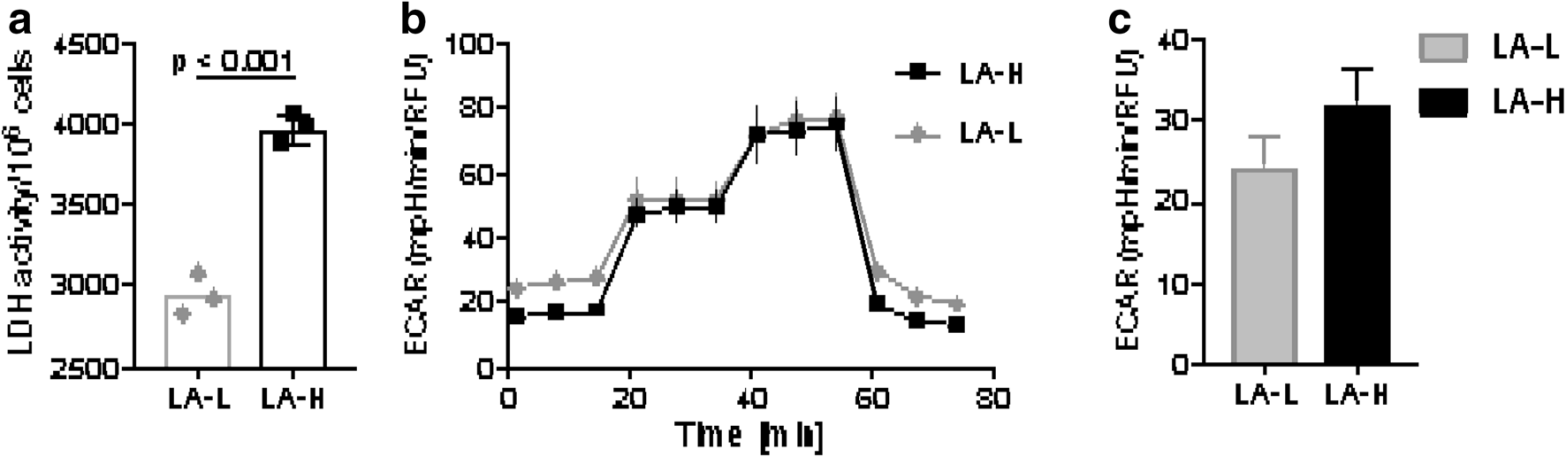
Significantly higher LDH activity was seen in LA-H cells compared to LA-L cells. Plots showing triplicate measurements of LA-H and LA-L cell lines in LDH activity (**a**). Mann–Whitney-U-test p < 0.001. Kinetics of ECAR (extracellular acidification rate) response in the *Seahorse Glycolysis stress* test assay (**b**) and calculated rate of basal glycolysis (**c**). RFU (relative fluorescence unit).

Cell extract LDH-activity assays revealed significantly higher LDH activity in LA-H cells (mean_LA-H_ 3971 ± 91 U/l, n = 3) compared to LA-L cells (mean_LA-L_ 2820 ± 125 U/l, n = 3; Mann–Whitney-U-test p < 0.001, Fig. 3a). The *Seahorse Glycostress* assay also indicated slightly elevated basal glycolysis in LA-H (32.3 ± 4.2 mpH/min/RFU) compared to LA-L cells (24.5 ± 3.8 mpH/min/RFU) (Fig. 3b, c).

**Figure 4:**
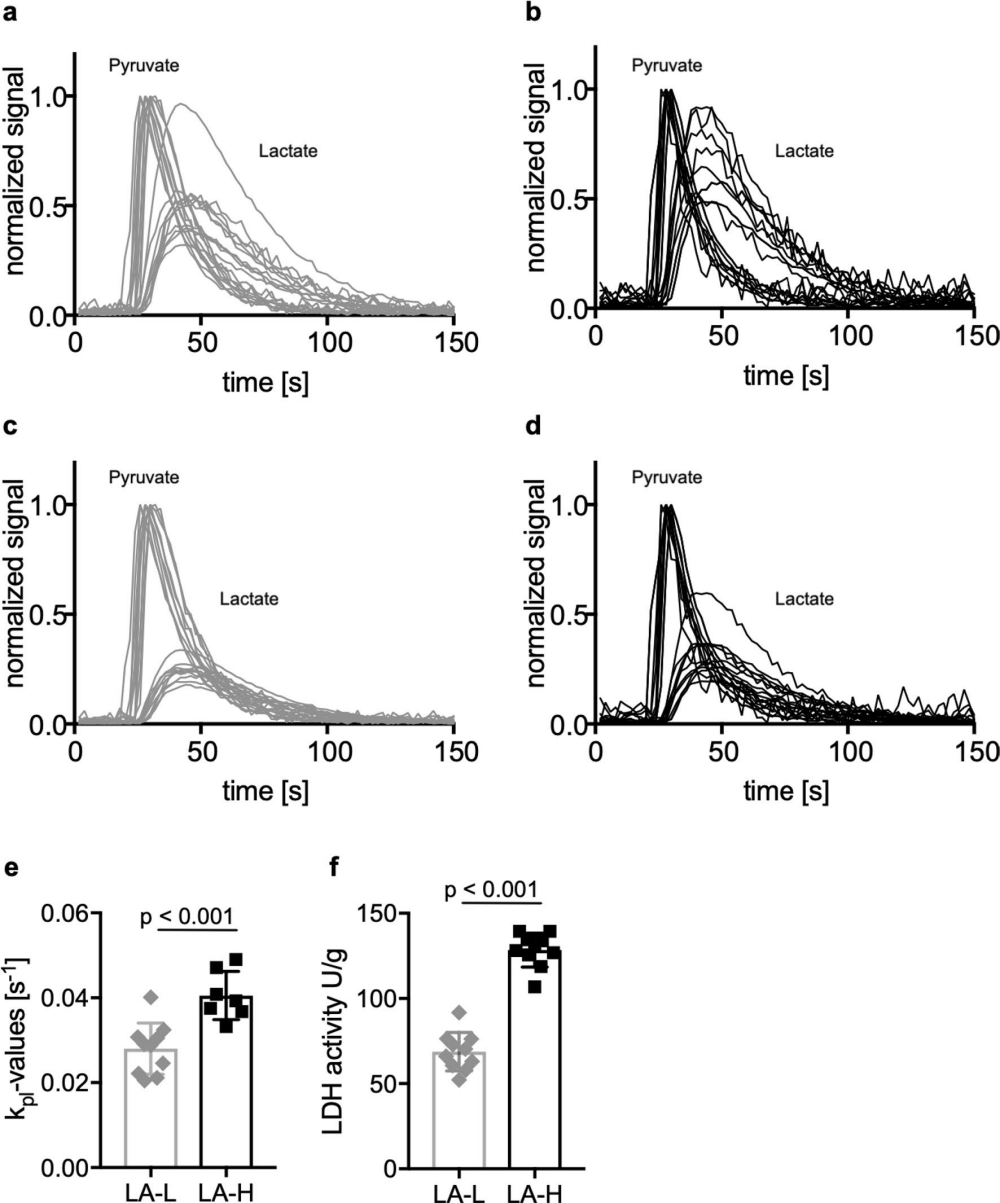
Normalized time-intensity curves of subcutaneous tumors derived from LA-L and LA-H cell lines. [1-^13^C]lactate and [1-^13^C]pyruvate signal curves of LA-L (**a**, **c**) and LA-H (**b**, **d**) tumors before (**a**, **b**) and after (**c**, **d**) application of the kinetic-model-derived scaling factors to the [1-^13^C]lactate signal curves. Metabolite intensities are normalized to pyruvate peak intensity. *k*_*p*l_-values of the same LA-L and LA-H tumors (**e**) and corresponding LDH activity of their extracts (**f**). Both Student’s T-test p < 0.001.

Characterized LA-L and LA-H cell line clones were successfully reestablished as subcutaneous tumors in nude rats. Tumors from LA-H cells reached ≥ 10 mm diameter faster (14.8 days) than tumors from LA-L cells (16.8 days). Visual inspection of slice-selective HPMRS revealed sufficient [1-^13^C]pyruvate and [1-^13^C]lactate signal to noise ratios. (Fig. 4a, b). To distinguish between tumor-derived (i.e. endogenous) and systemic (i.e. wash-in) lactate and to allow the computation of accurate exchange rate constants between [1-^13^C]pyruvate and [1-^13^C]lactate (*k*_*pl*_), a two-site exchange model was employed, with scaling factors applied to the [1-^13^C]lactate signal time-course (on average for all tumors 0.48; range: 0.35 to 0.85, see Supplementary Figures S5 and S6) such that the fit *T*_1_ of [1-^13^C]pyruvate was fixed at 30 s. In detail, [1-^13^C]lactate signal curves were iteratively multiplied with scaling factors between 0 and 1 in steps of 0.05 and the resulting lactate signal curve was then fitted with the kinetic model. The fit for which T_1,pyr_ closest matched *T*_1,pyr_ = 30 s was chosen to be valid for *k*_pl_. Scaled data, shown in Fig. 4c,d, revealed significantly higher *k*_*pl*_ values for LA-H compared to LA-L derived tumors (0.041 ± 0.008 s^-1^ vs. 0.028 ± 0.01 s^-1^, p < 0.001) (Fig. 4e). Three *k*_*pl*_ values were excluded because of a fit residual > 0.7.

Ex vivo LDH activity measurement of the tumor tissue extracts corroborated this finding by demonstrating significantly higher LDH activity in LA-H tumor extracts compared to LA-L extracts (128 ± 16 U/g vs. 69 ± 20 U/g, Student’s T-test p < 0.001, Fig. 4f).

No differences were noted in immunohistochemically assessed vascularization. However, LA-H tumors showed predominantly vacuolized tumor cells compatible with lipid accumulation (fatty change) and only few solid tumor areas, whereas LA-L tumors showed larger solid areas of neoplastic cells with an eosinophilic cytoplasm, sometimes with a spindle cell appearance interspersed with vacuolized tumor cells (see Supplementary Figure S7).

## Discussion

Here we introduce the non-invasive metabolic phenotyping of hepatocellular carcinoma (HCC) by HPMRS(I) in endogenous and reimplanted subcutaneous rat tumor models. Our results constitute a proof-of-concept for HPMRS(I) in HCC and provide a framework for a better understanding of in vivo metabolic processes underlying pathophysiology and options for treatment. The here-established rat HCC cell lines with differing lactate production will allow further dissection of the underlying genetic tumor landscape.

HPMRS(I) provides additional insight into in vivo metabolic activity beyond ^18^F-FDG PET imaging, a standard clinical functional imaging method, as it allows for the real time visualization of enzymatic conversions^26^. Pyruvate is a downstream metabolite of glycolysis that can be used to differentiate tissues based on the predominant enzymatic reaction leading towards the production of lactate, alanine or utilization in the TCA cycle. Lactate provides growth advantages to tumors like immune evasion and promotion of angiogenesis^3,5,6^, and it has been shown in other tumor entities that high lactate production leads to higher biological aggressiveness^7–9^, mirrored in the higher growth rates observed in LA-H tumors. Furthermore, pyruvate can be a promising probe molecule for the differentiation of particularly aggressive tumor phenotypes, as has been shown for several tumor entities, including breast cancer^27^ or for the early monitoring of tumor cell death, where HPMRS has been shown to outperform ^18^F-FDG PET^28^.

In human HCC, LDH activity is up-regulated as a result of miRNA383 suppression^29^ and serum LDH levels have shown a prognostic value in the prognostication of HCC treatment response^30,31^. Additionally, a shift towards increased lactate production was described in human HCC tissue samples^32^. Furthermore, the suppression of MCT in human HCC cells resulted in a decrease of the tumor proliferation rate^33^. Thus, the non-invasive monitoring of glycolysis and in particular the alternative conversion of pyruvate into lactate could serve as an indicator of therapy response to novel metabolic therapy interventions such as PARP inhibition by Olaparib^11^.

In vivo MRS offers options beyond pyruvate with additional substrates such as glutamate^34^ or zymonic acid^35^, which can be used for probing of alternate metabolic pathways such as oxidative phosphorylation, pH or inflammation-related phenomena. A downregulation of oxidative phosphorylation with a concurrent increase in inflammation has recently been shown to be associated with significant differences in HCC patient survival^10^. Our own results also show an increased extracellular acidification in LA-H cells in the in vitro* Seahorse* assay, supporting this finding.

However, major technical and logistic challenges like low SNR, short signal half-life and rapid signal decay due to applied radiofrequency pulses pose strong limitations to the experimental setup and must be overcome to enable clinical usage of HPMRSI. Until these barriers are resolved, predictive model systems such as the here-employed endogenous^19^ and subcutaneous rat models are a necessity and can bridge the translational gap on the molecular level for differentiation of metabolic heterogeneity and testing of personalized therapy regimens, e.g. via patient derived xenograft model systems^36^.

A further challenge in the extraction of quantitative measures arises from the cellular export and re-circulation of lactate, mandating the inclusion of systemic lactate levels into the modelling of local conversion rates^37^. For example, Wespi et al. demonstrate that ^13^C-lactate signal of the heart measured by slice selective spectroscopy is overestimated because of contributions from the liver to the ^13^C-lactate signal of the heart^38^. Lactate wash-in can originate from label exchange in other organs or other tumor sites^37^. In this work, the additional lactate inflow term was estimated and accounted for using a two-site kinetic exchange model with a scaling factor separating wash-in [1-^13^C]lactate from the locally originating tumor [1-^13^C]lactate signal. The scaling factor was determined by matching the fitted *T*_1_ of [1-^13^C]pyruvate to its previously reported value for in vivo experiments, i.e. 30 s^22,39^.

As a potential limitation of our study, isolated cell clones might have undergone cell culture-related selection processes which cannot be adequately distinguished in the imaging experiments, although cell morphology is consistent with previous findings^40^. Additionally, subcutaneous tumor generation does not represent the normal process of hepatocarcinogenesis, nor the microenvironment of the liver, both of which are better represented in the endogenous tumor model we employed. The subcutaneous model however allows a precise positioning of surface coils for measurements. Furthermore, we believe the influence of macro-metabolism to still be unclear, although we followed recommendations regarding fasting the animals before image acquisition^41^. Lastly, in vivo imaging techniques employed in our study suffer from the limitation of low spatial resolution, requiring further technical developments.

In conclusion, our study provides evidence for the usage of HPMRS(I) in the in vivo metabolic differentiation of HCC. The application of HPMRS(I) in preclinical therapy trials will enable the validation of HPMRS(I)-based endpoints as surrogates of tumor biology and therapy response.

## Supplementary information

Supplementary Information.
