# Author Correction: Hyperpolarized ^13^C pyruvate magnetic resonance spectroscopy for in vivo metabolic phenotyping of rat HCC

**DOI:** 10.1038/s41598-021-90697-3

**Published:** 2021-05-28

**Authors:** Elisabeth Bliemsrieder, Georgios Kaissis, Martin Grashei, Geoffrey Topping, Jennifer Altomonte, Christian Hundshammer, Fabian Lohöfer, Irina Heid, Dominik Keim, Selamawit Gebrekidan, Marija Trajkovic-Arsic, AM Winkelkotte, Katja Steiger, Roman Nawroth, Jens Siveke, Markus Schwaiger, Marcus Makowski, Franz Schilling, Rickmer Braren

**Affiliations:** 1 School of Medicine, Institute of Diagnostic and Interventional Radiology, Technical University of Munich, 81675 Munich, Germany; 2 School of Medicine, Department of Nuclear Medicine, Technical University of Munich, 81675 Munich, Germany; 3 School of Medicine, Clinic and Policlinic of Internal Medicine II, Technical University of Munich, 81675 Munich, Germany; 4 Division of Solid Tumor Translational Oncology, German Cancer Consortium (DKTK, Partner Site Essen) and German Cancer Research Center, DKFZ, Heidelberg, Germany; 5 Institute for Developmental Cancer Therapeutics, West German Cancer Center, University Medicine Essen, 45147 Essen, Germany; 6 School of Medicine, Institute of Pathology, Technical University of Munich, 81675 Munich, Germany; 7 School of Medicine, Clinic and Policlinic of Urology, Technical University of Munich, 81675 Munich, Germany; 8 Department of Computing, Imperial College London, London, SW7 2AZ UK

Correction to: *Scientific Reports* 10.1038/s41598-020-80952-4, published online 13 January 2021

The original version of this Article contained an error in the spelling of the author AM Winkelkotte which was incorrectly given as Aline Winkelkotte.

In addition, the Funding section was incomplete.

“Open Access funding enabled and organized by Projekt DEAL.”

now reads:

“This work was supported by the Deutsche Forschungsgemeinschaft (DFG, German Research Foundation—391523415, SFB 824) to Rickmer Braren and Franz Schilling. Georgios Kaissis received funding from the Technical University of Munich clinician scientist programme (Grant Reference H14). Jens Siveke is supported by the German Cancer Consortium (DKTK), the Deutsche Forschungsgemeinschaft (DFG, Collaborative Research Center SFB824 (project C4, INST 95/1017-3), SI 1549/3-1 (Clinical Research Unit KFO337), SI 1549/4-1 / AI 186/1) and the German Cancer Aid (grant no. 70112505; PIPAC consortium) and reports research funding from BMS, Celgene and Roche; consulting and personal fees from AstraZeneca, Bayer, Baxalta, BMS, Celgene, Immunocore, Lilly, Novartis, Roche, Shire; has minor equity in FAPI Holding and Pharma15 (<3%) and is a member of the Board of Directors for Pharma15 outside the submitted work. Open Access funding enabled and organized by Projekt DEAL.”

The original Article has been corrected.

