# Supplementary material for: Hyperpolarized 13C pyruvate magnetic resonance spectroscopy for in vivo metabolic phenotyping of rat HCC
Source: Sci Rep. 2021 Jan 13;11:1191. doi: 10.1038/s41598-020-80952-4 (PMC7806739; doi:10.1038/s41598-020-80952-4)
Supplement: Supplementary file 1 — Supplementary Information. [file 41598_2020_80952_MOESM1_ESM.docx]

Hyperpolarized [1-^13^C]pyruvate magnetic resonance spectroscopy for *in vivo* metabolic phenotyping of rat HCC

Bliemsrieder E.*^1,2^, Kaissis GA.*^1, 8^, Grashei M. ^2^, Topping GJ. ^2^, Altomonte J. ^3^, Hundshammer C. ^2^, Lohöfer FK. ^1^, Heid I. ^1^, Keim D. ^1^, Gebrekidan S. ^1^, Trajkovic-Arsic M. ^4,5^, Winkelkotte AM. ^4,5^, Steiger K. ^6^, Nawroth R. ^7^, Siveke J. ^4,5^, Schwaiger M. ^2^, Makowski M.R. ^1^, Schilling F. ^2^, Braren RF^1^.

* equally contributing authors

**Affiliations**

^1^ Technical University of Munich, School of Medicine, Institute of Diagnostic and Interventional Radiology, 81675 Munich, Germany

^2^ Technical University of Munich, School of Medicine, Department of Nuclear Medicine, 81675 Munich, Germany

^3^ Technical University of Munich, School of Medicine, Clinic and Policlinic of Internal Medicine II, 81675 Munich, Germany

^4^ Division of Solid Tumor Translational Oncology, German Cancer Consortium (DKTK, partner site Essen) and German Cancer Research Center, DKFZ, Heidelberg, Germany

^5^ Institute for Developmental Cancer Therapeutics, West German Cancer Center, University Medicine Essen, 45147 Essen, Germany

^6^ Technical University of Munich, School of Medicine, Institute of Pathology, 81675 Munich, Germany

^7^ Technical University of Munich, School of Medicine, Clinic and Policlinic of Urology, 81675 Munich, Germany

^8^ Imperial College London, Department of Computing, SW7 2AZ London, United Kingdom

Corresponding author e-mail: rbraren@tum.de

## Supplementary Material

**^1^H-NMR spectroscopy**

To quantify lactate and alanine in the extracts of endogenous tumors, the integral over frequency of the spectral peak of 0.01 mM hydrochinone in a separate NMR tube under equal solvent and acquisition conditions was used as a reference and compared with the integrals of lactate and alanine. The integral of 0.01 mM hydrochinone was defined as unity. The integral of a proton NMR spectral peak magnitude signal is proportional to the proton concentration. As hydrochinone carries 4 protons and lactate and alanine carry 3, for integrals of the same area, the resulting concentration of lactate and alanine is 1.33 times the concentration of hydrochinone.

**
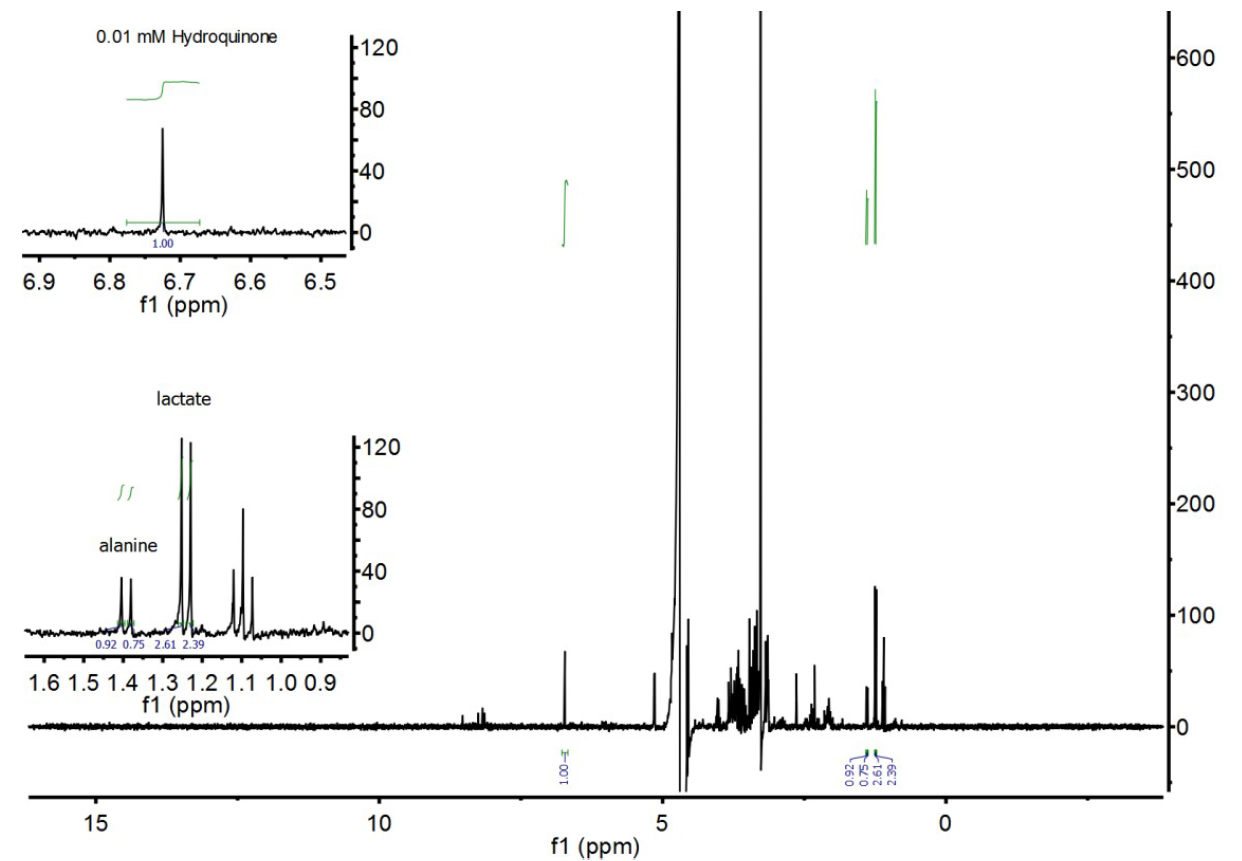
**

Supplementary Figure S1. Sample dataset showing the overview of a ^1^H NMR spectrum (right) and a zoomed spectrum on hydrochinone (top left panel; δ = 6.73 ppm) as well as a zoomed spectrum of the lactate and alanine region (bottom left; doublet lactate δ = 1.25 ppm; doublet alanine δ = 1.4 ppm).

*Calculated integral position: blue; range used for calculation: green; ppm: parts per million*

**Details of metabolite fitting**

Time curves were derived from peak maxima of the respective metabolite in the magnitude spectrum for each time point. The resulting time curves were baseline-corrected and both curves were fitted starting from the time point of the pyruvate maximum. Fitting of the original metabolite time curves (Figure S2a) yields a corresponding spin‑lattice relaxation time constant of [1-^13^C]pyruvate *T*_1,pyr_ = ‑26.34 s as calculated from eq. 1 and

$\frac{1}{T_{1,pyr}}=R_{pyr,eff}- k_{pl}$ .

Such a negative *T*_1,pyr_ is physically not plausible. This indicates more lactate production than theoretically possible. Intersect of the metabolite curves at normalized intensities > 0.5 validate this overestimated lactate production, even in the case of no relaxation effects, which can be explained by wash-in from other regions outside of the tumor. Here, this is accounted for by modelling two overall lactate signal fractions with equal dynamics, or namely curve shape, for inflowing systemic and intratumorally produced lactate. In this model, the intratumorally produced lactate can be derived by multiplying the original signal curve with a scaling factor between 0 and 1 (Figure S2b, FigureS4)*.* This factor is chosen such that subsequent fitting of the original pyruvate and the scaled lactate time curve produce *T*_1,pyr_ = 30 s within the discrete step size of 0.05. In detail, the curve is first iteratively scaled with a scaling factor ranging from 0 to 1 and the resulting lactate curve is then fitted together with the pyruvate signal curve. After correcting the resulting *T*_1,pyr_ for repeated excitation (flip angle correction), the scaling factor matching closest *T*_1,pyr_ = 30 s is chosen and the corresponding kpl-value used. The resulting values of the scaling factor ranged from 0.35 to 0.85, varying between tumors and animals, and allowed the tumor phenotypes to be better distinguished based on *k*_pl_ rate constants.

**
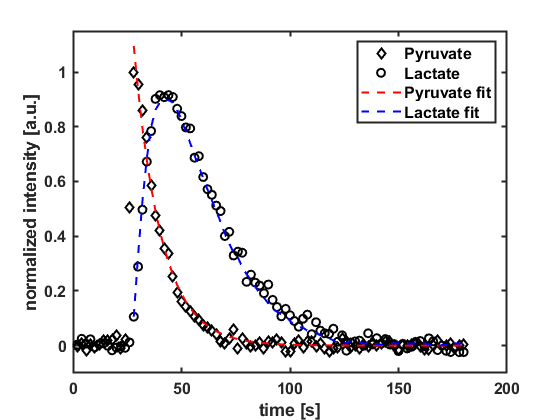
**
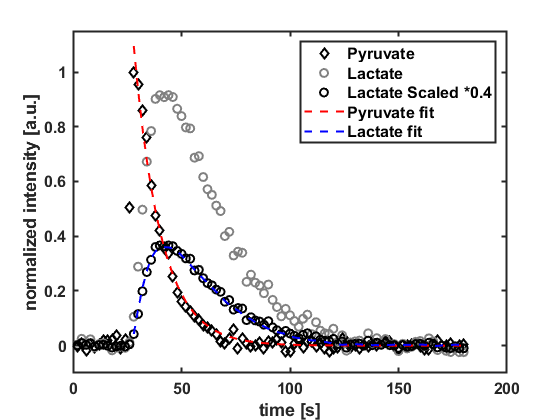


Supplementary Figure S2. Unscaled (a) and scaled (b) [1‑^13^C]lactate and (unscaled) [1‑^13^C]pyruvate time curves. Dynamic pyruvate- and lactate-signal intensity curves were fitted with a kinetic two-site-exchange model. Fitting of the unscaled time curves (A) results in an implausible value for *T*_1,pyr_ = -26.34 s and a consequently overestimated *k*_pl_ = 0.118 $\frac{1}{s}$ . Correction of the lactate time curve to be closest to *T*_1,pyr_ = 30 s and subsequent fitting (B) returns a *k*_pl_ = 0.047 $\frac{1}{s}$ .

**Differences in cell and tissue morphology between LA-H and LA-L tumors**

Cell lines derived from LA-L and LA-H tumors displayed differences in cell shape:

Supplementary Figure S3. Cell morphology of LA-L and LA-H cell lines in 2D *in vitro* culture. Light microscopy images of LA-L (a) and LA-H (b) cell lines.

**Details on Scaling Factor**

Supplementary Figure S4. Scaling factors derived from kinetic modeling. While values strongly scatter from 0.35 to 0.85, assignment to the corresponding tumor phenotypes shows no significant difference between the groups (LA-L: 0.49±0.04 vs. LA-H: 0.50±0.04, both n=10, Mann-Whitney-U-test p = 0.80).

Scaling factors for each lactate time curve were derived as discrete multiples of 0.05 from kinetic modeling as described above with the correct value being closest to *T*_1,pyr_ = 30 s after flip angle correction. Values of both groups ranging from 0.35 to 0.85 demonstrate that the amount of lactate being produced in the tumor can represent less than half of the overall measured lactate signal. While exhibiting strong heterogeneity of the values being calculated for each phenotype, no significant difference between the groups can be observed.


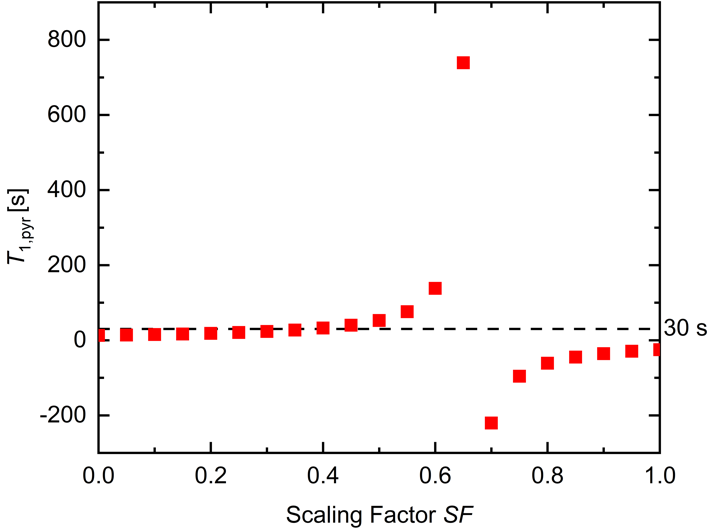

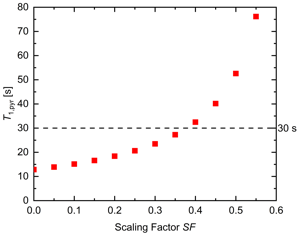

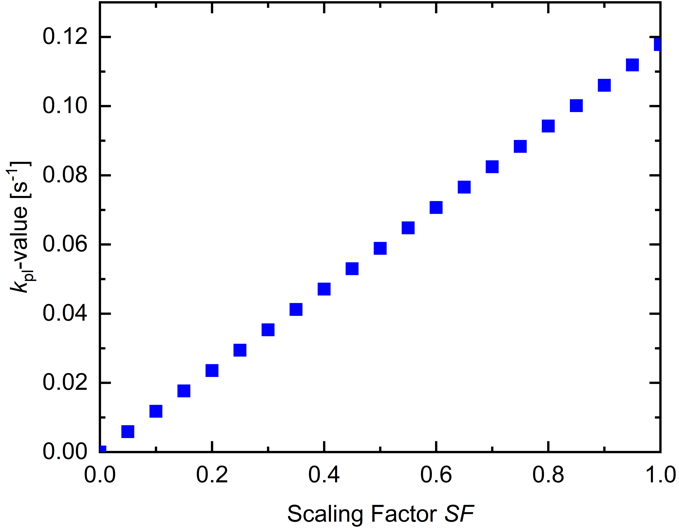


**a**

**b**

Supplementary Figure S5. Dependencies of flip angle corrected *T*_1,pyr_ (a) and *k*_pl_ (b) on the scaling factor *SF.* The reciprocal relation between *T*_1,pyr_ and *SF* allows an accurate determination of the appropriate value for *SF* regarding the step-size of 0.05 while small errors in *SF* propagate linearly to *k*_pl_ values. The data shown in this plot is of the same subject as the plots shown in Figure S1. Inset in (a): Zoom of the region where curve intersects *T*_1,pyr_ = 30s.

As the dependency on SF is reciprocal for *T*_1,pyr_ and linear for *k*_pl_ (Figure S5a), this allows to accurately determine the correct value *SF* on the discretization scale of 0.05. In addition, small errors in *SF* only lead to small inaccuracies of *k*_pl_ due to the linear dependency between both (Figure S5b). Taking this into account, it is still possible to reliably separate the systemic lactate production from the tumor lactate and therefore quantify tumor metabolism. In order to further evaluate and improve the introduced model, future work could assess the reliability of this model for tumors producing less or almost no lactate by simulations or approaches to measure the tumor inflow characteristics for systemic lactate, as already shown in other work^1^. Also, as in the current study animals were always measured bearing two subcutaneous tumors of the same phenotype/cell line, comparisons to animals bearing only one tumor might reveal further information about lactate recirculation.

**H&E staining of LA-L and LA-H tumors displayed differences in tissue morphology**


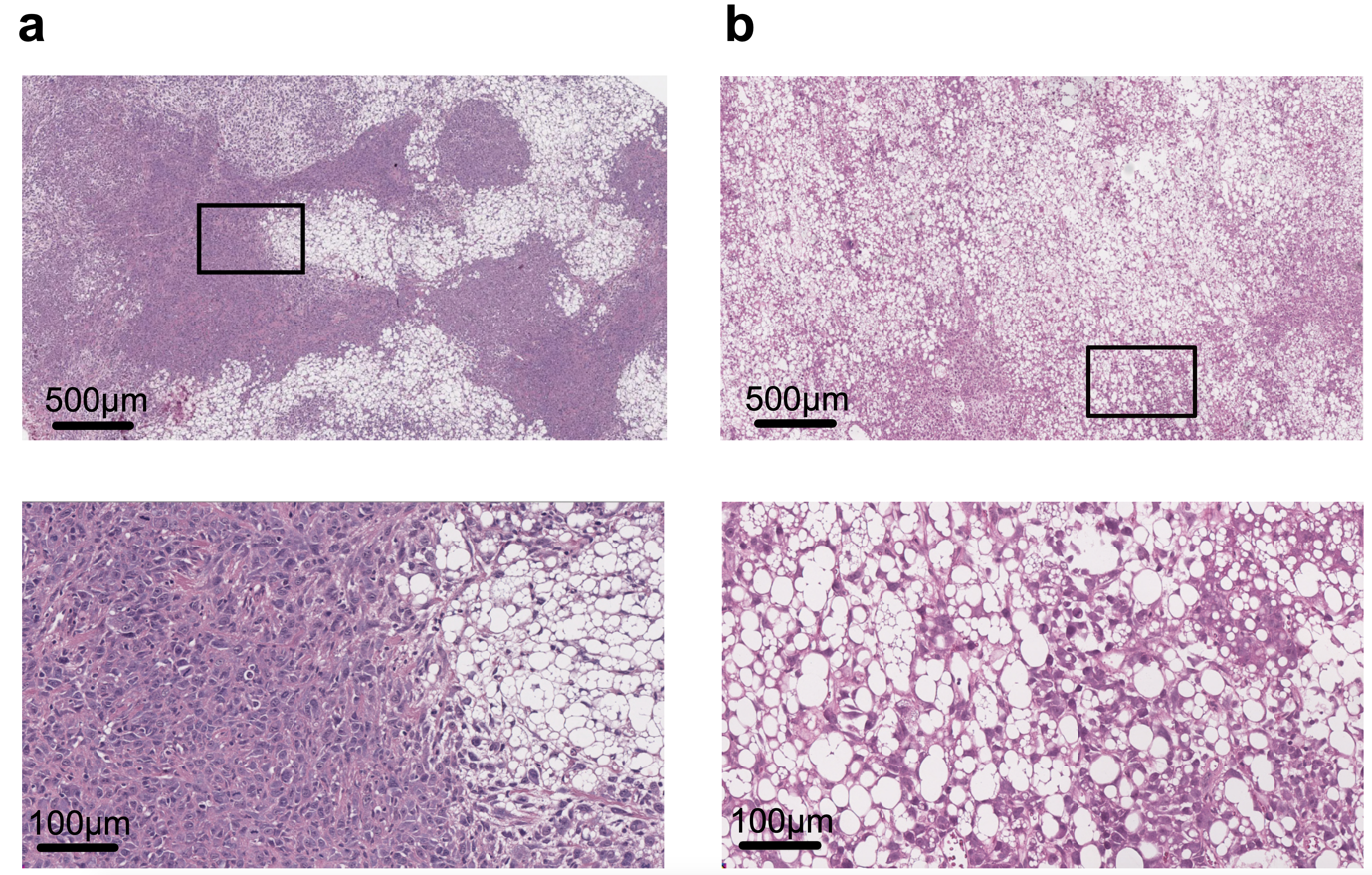


Supplementary Figure S6. Histopathology of LA-H and LA-L tumors. Light microscopy images of H&E staining of LA-L (a) and LA-H (b) tumors. Top low magnification overview, bottom higher magnification inset.

**Supplementary Reference**

1. Kazan, S.M., Reynolds, S., Kennerley, A., Wholey, E., Bluff, J.E., Berwick, J., Cunningham, V.J., Paley, M.N. and Tozer, G.M. (2013), Kinetic modeling of hyperpolarized 13C pyruvate metabolism in tumors using a measured arterial input function. Magn. Reson. Med., 70: 943-953. doi:10.1002/mrm.24546
